# What health are we talking about? Biodiversity as the missing link between One Health and Planetary Health

**DOI:** 10.1007/s13280-026-02487-8

**Published:** 2026-09-15

**Authors:** Jonas Geschke, Simone Sommer, Michael Schloter

**Affiliations:** 1 Institute of Plant Sciences, University of Bern, Altenbergrain 21, 3013 Bern, Switzerland; 2 Institute of Evolutionary Ecology and Conservation Genomics, University of Ulm, Albert-Einstein-Allee 11, 89081 Ulm, Germany; 3 Chair of Environmental Microbiology, Technical University of Munich, Email-Ramann-Straße 2, 85354 Freising, Germany

**Keywords:** Biodiversity, Ecosystem health, One Health, Planetary Health, Public health, Sustainability policy

## Abstract

Health has become a central term in global sustainability policy, yet it is often used without sufficient conceptual precision. Public health, global health, One Health, EcoHealth, GeoHealth, and Planetary Health each emphasize different dimensions of the relationship between humans, animals, and the environment. In policy contexts, however, these distinctions are frequently blurred. We argue that biodiversity is often treated as an environmental co-benefit rather than as a foundational determinant of health. This weakens the implementation of One Health approaches because biodiversity underpins disease regulation, immune system development, food and water security, ecosystem functioning, resilience, and climate adaptation. At the same time, biodiversity provides a critical link between One Health and broader Planetary Health challenges, including global environmental change and the transgression of planetary boundaries. Future health and sustainability policies should move beyond generic references to health and explicitly recognize biodiversity as part of preventive health systems.

## Introduction

Health has become a guiding concept in global environmental and sustainability policy, yet its understanding differs substantially across scientific disciplines and policy arenas. In biomedical contexts, health is often approached through disease, therapy, and individual well-being. Public and global health shift attention toward populations, prevention, and governance. The One Health approach emphasizes the interdependence of human, animal, and environmental health, whereas Planetary Health places human well-being within the stability of Earth systems. Related approaches, such as EcoHealth and GeoHealth, further broaden the perspectives by emphasizing ecological and environmental processes. Although these concepts overlap, each has distinct theoretical foundations and practical implications and they should not be regarded as interchangeable (WBGU 2023; Müller et al. 2025).

International policy frameworks generally treat health as a broad societal objective rather than adopting specific health concepts. The Paris Agreement identifies health as a key concern affected by climate change (UN 2015a) but does not endorse a particular framework. Similarly, the 2030 Agenda for Sustainable Development addresses health through Sustainable Development Goal 3 and integrates it across multiple goals (UN 2015b), yet without distinguishing among different health concepts. The Kunming-Montreal Global Biodiversity Framework is a notable exception, explicitly referencing the One Health approach as a consideration for its implementation and recognizing links between biodiversity, wildlife, and disease risks (CBD 2022).

Overall, international agreements use health as an inclusive, cross-cutting objective while avoiding detailed conceptual distinctions. This ambiguity matters because concepts shape implementation: A narrow focus on disease prevention may treat environmental degradation as an external risk, whereas broader approaches view ecosystem functioning and environmental stability as foundations of health. At the same time, despite increasing recognition of environmental determinants of health, biodiversity itself often remains insufficiently integrated into health policy and governance frameworks.

## Biodiversity as a link between One Health and Planetary Health

Biodiversity is often viewed primarily as an environmental issue, yet it is increasingly recognized as a fundamental determinant of health. Even within One Health, which seeks to balance and optimize the health of people, animals and ecosystems while recognizing their interdependence (OHHLEP 2022; One Health Commission 2026), biodiversity is frequently acknowledged but rarely placed at the center of implementation. This represents a missed opportunity. Biodiversity is not merely a conservation objective that exists alongside health; it is a fundamental biological foundation through which health is generated, maintained, and protected.

One of the most direct contributions of biodiversity to health is through the production of healthy and resilient food systems. Soil biodiversity, including microbial communities and plant diversity, pollinators, and natural enemies of pests underpin soil fertility, nutrient cycling, crop productivity, plant health, and dietary diversity (Soliveres et al. 2016; Schloter et al. 2017; Bender et al. 2023; Jetter et al. 2025). These ecological processes influence micronutrient availability, reduce dependence on pesticides and antimicrobials, enhance the resilience of food production under climate stress, and ultimately improve food quality (Schloter and Schulz 2025). Biodiversity therefore provides a direct link between ecosystem functioning, nutrition, food security, and disease prevention (IPBES 2024).

Biodiversity also influences infectious disease dynamics by shaping pathogen transmission, host competence, and interactions among humans, livestock, wildlife, and their shared environments (Sessitsch et al. 2023; Meyer et al. 2024, 2025). Biodiversity loss, deforestation, land-use change, and agricultural intensification can disrupt ecological relationships and create new opportunities for pathogen spillover, although these effects are highly context dependent (Faust et al. 2018; Gibb et al. 2020; IPBES 2020). Rather than supporting the simplistic assumption that higher biodiversity always reduces disease risk, current evidence indicates that disease dynamics emerge from complex ecological systems in which biodiversity plays important regulatory roles, particularly through host–microbe interactions and trophic networks (Soliveres et al. 2016; Fackelmann et al. 2021; Sessitsch et al. 2023; Fleischer et al. 2024a, b). These findings highlight the importance of maintaining ecological integrity as part of disease prevention and health system resilience.

Beyond infectious diseases, biodiversity also contributes directly to immune development through exposure to diverse environmental microbiota. Contact with biodiverse soils, plants, animals, and outdoor environments enriches the diversity of host-associated microbial communities and supports the development of immune tolerance, particularly during early life (Roslund et al. 2020; Marques Dos Santos et al. 2023). Conversely, biodiversity loss, urbanization, reduced contact with natural environments, and the homogenization of microbial exposures can contribute to immune dysregulation and increase susceptibility to allergies, asthma, and other chronic inflammatory diseases (Birzele et al. 2017).

Microbial diversity provides a key mechanistic connection across the dimensions of One Health. Microbiomes are integral components of humans, animals, plants, soils, freshwater and marine systems, and the built environment, mediating interactions between environmental conditions and biological functioning. Changes in microbiome composition can serve as sensitive indicators of environmental and health-related stress, while microbial diversity contributes to resilience, stability, and adaptive capacity across biological systems (Schloter et al. 2017; Wasimuddin et al. 2018, 2019; Berg et al. 2020; Alpízar et al. 2021; Fackelmann et al. 2021; Risely et al. 2023; Sessitsch et al. 2023). Recognizing microbial diversity as a shared component of environmental, animal, and human health therefore strengthens the conceptual and operational integration of the One Health approach.

Viewed across scales—from microbial communities in soils to ecosystems spanning continents and oceans—biodiversity underpins the ecological processes that sustain life. In doing so, it provides the biological foundation that links One Health with Planetary Health, emphasizing that safeguarding biodiversity is not only an environmental priority but also an essential strategy for protecting the health and resilience of present and future generations.

## From conceptual clarity to implementation

Recognizing biodiversity as foundational to health is not merely a conceptual shift but has important implications for policy, governance, and implementation. Rather than choosing between One Health and Planetary Health, these frameworks should be viewed as complementary. One Health provides an operational framework for collaboration across medicine, veterinary science, agriculture, and environmental management, and Planetary Health emphasizes the ecological boundaries within which societies and health systems operate. Biodiversity connects these perspectives by linking local ecological processes with global environmental change.

Translating this understanding into practice requires integrating biodiversity into health planning, pandemic preparedness, veterinary strategies, and antimicrobial resistance action plans. Surveillance should extend beyond human and livestock health to include wildlife, environmental, and ecosystem indicators, while institutions across health, agriculture, environment, finance, and urban planning should develop shared data systems and joint risk assessments. Ecosystem conservation and restoration should be recognized as investments in prevention and resilience, and research should further explore microbiomes, ecosystem services, and nature-based solutions as mechanisms linking biodiversity and health (Sessitsch et al. 2023; Schröder et al. 2024).

Agriculture, food systems, and urban environments are central to this transition. Agriculture is a major driver of biodiversity loss but also shapes nutrition, livelihoods, antimicrobial use, and climate resilience. Likewise, cities concentrate environmental health risks while offering opportunities to improve access to biodiverse green spaces and healthier food systems. Biodiversity-friendly agriculture, regenerative food systems, urban green infrastructure, healthy soils, wetlands, community gardens, and other nature-based solutions can simultaneously strengthen biodiversity, food security, climate adaptation, and physical and mental health (WBGU 2023; IPBES 2024). Together, these transformations offer substantial co-benefits across human, animal, environmental, and planetary health.

To conclude, the question *“What health are we talking about?”* is more than semantic. It determines how problems are framed, which sectors engage, and which interventions are prioritized. It also supports a shift from disease treatment toward prevention and health promotion, with important socioeconomic implications for health systems and resource allocation. Although current policy debates increasingly acknowledge the importance of health, they often fail to explicitly connect human, animal, ecosystem, and planetary dimensions. We argue that biodiversity should be recognized as a foundational determinant of health and as the critical link across these dimensions. Future sustainability frameworks should therefore move beyond treating health as a generic umbrella term and instead adopt a conceptually differentiated understanding that places biodiversity at the center of healthy people, ecosystems, and the planet.

## References

[CR1] Alpízar, P., A. Risely, M. Tschapka, and S. Sommer. 2021. Agricultural fast food: Bats feeding in banana monocultures are heavier but have less diverse gut microbiota. *Frontiers in Ecology and Evolution* 9: 746783. 10.3389/fevo.2021.746783.

[CR2] Bender, S.F., S. Schulz, R. Martinez-Cuesta, R.J. Laughlin, S. Kublik, K. Pfeiffer-Zakharova, G. Vestergaard, K. Hartman, et al. 2023. Simplification of soil biota communities impairs nutrient recycling and enhances above- and belowground nitrogen losses. *New Phytologist* 240: 2020–2034. 10.1111/nph.19252. 37700504

[CR3] Berg, G., D. Rybakova, D. Fischer, T. Cernava, M.C. Verges, T. Charles, X. Chen, L. Cocolin, et al. 2020. Microbiome definition re-visited: Old concepts and new challenges. *Microbiome* 8: 103. 10.1186/s40168-020-00875-0. PMC7329523 32605663

[CR4] Birzele, L.T., M. Depner, M.J. Ege, M. Engel, S. Kublik, C. Bernau, G.J. Loss, J. Genuneit, et al. 2017. Environmental and mucosal microbiota and their role in childhood asthma. *Allergy* 72: 109–119. 10.1111/all.13002. 27503830

[CR5] CBD-Convention on Biological Diversity. 2022. *Kunming-Montreal Global Biodiversity Framework*. *CBD/COP/DEC/15/4*.

[CR6] Fackelmann, G., M.A.F. Gillingham, J. Schmid, A.C. Heni, K. Wilhelm, N. Schwensow, and S. Sommer. 2021. Human encroachment into wildlife gut microbiomes. *Communications Biology* 4: 800. 10.1038/s42003-021-02315-7. PMC8233340 34172822

[CR7] Faust, C.L., H.I. McCallum, L.S.P. Bloomfield, N.L. Gottdenker, T.R. Gillespie, C.J. Torney, A.P. Dobson, and R.K. Plowright. 2018. Pathogen spillover during land conversion. *Ecology Letters* 21: 471–483. 10.1111/ele.12904. 29466832

[CR8] Fleischer, R., G.J. Eibner, N.I. Schwensow, F. Pirzer, S. Paraskevopoulou, G. Mayer, V.M. Corman, C. Drosten, et al. 2024a. Immunogenetic-pathogen networks shrink in Tome’s spiny rat, a generalist rodent inhabiting disturbed landscapes. *Communications Biology* 7: 169. 10.1038/s42003-024-05870-x. PMC10858909 38341501

[CR9] Fleischer, R., C. Jones, P. Ledezma-Campos, G.Á. Czirják, S. Sommer, T.R. Gillespie, and A. Vicente-Santos. 2024b. Gut microbial shifts in vampire bats linked to immunity due to changed diet in human disturbed landscapes. *Science of the Total Environment* 907: 167815. 10.1016/j.scitotenv.2023.167815. 37852483

[CR10] Gibb, R., D.W. Redding, K.Q. Chin, C.A. Donnelly, T.M. Blackburn, T. Newbold, and K.E. Jones. 2020. Zoonotic host diversity increases in human-dominated ecosystems. *Nature* 584: 398–402. 10.1038/s41586-020-2562-8. 32759999

[CR11] IPBES-Intergovernmental Science-Policy Platform on Biodiversity and Ecosystem Services. 2020. *Workshop report on biodiversity and pandemics of the Intergovernmental Science-Policy Platform on Biodiversity and Ecosystem Services*.

[CR12] IPBES-Intergovernmental Science-Policy Platform on Biodiversity and Ecosystem Services. 2024. *Summary for policymakers of the thematic assessment of the interlinkages among biodiversity, water, food and health of the Intergovernmental Science-Policy Platform on Biodiversity and Ecosystem Services*.

[CR13] Jetter, K., K. Jani, K. Wilhelm, U. Stehle, R.R. Chamedjeu, C.U. Riedel, L. Wilfert, P. Schafer, et al. 2025. Fertilization impacts microbiomes along the grassland trophic chain. *ISME Communications* 5: ycaf162. 10.1093/ismeco/ycaf162. PMC12503161 41064046

[CR14] Marques Dos Santos, M., V. Pivniouk, B. Rankl, A. Walker, G. Pagani, N. Hertkorn, P. Schmitt-Kopplin, C. Muller, et al. 2023. Asthma-protective agents in dust from traditional farm environments. *Journal of Allergy and Clinical Immunology* 152: 610–621. 10.1016/j.jaci.2023.05.013. PMC10680491 37271318

[CR15] Meyer, M., G. Eibner, A.C. Heni, K. Wilhelm, and S. Sommer. 2025. Changes in biodiversity drive trypanosome infections of wildlife in Panama. *One Health* 21: 101113. 10.1016/j.onehlt.2025.101113. PMC12226096 40612686

[CR16] Meyer, M., D.W. Melville, H.J. Baldwin, K. Wilhelm, E.E. Nkrumah, E.K. Badu, S.K. Oppong, N. Schwensow, et al. 2024. Bat species assemblage predicts coronavirus prevalence. *Nature Communications* 15: 2887. 10.1038/s41467-024-46979-1. PMC10994947 38575573

[CR17] Müller, V., C.-J. Sonntag, A. Seidler, and A. Freiberg. 2025. Health concepts in the context of anthropogenic environmental change: A scoping review on One Health, Planetary Health, EcoHealth, Public Health, Urban Health, and Global Health. *Journal of Public Health*. 10.1007/s10389-025-02659-y.

[CR18] OHHLEP-One Health High-Level Expert Panel, W.B. Adisasmito, S. Almuhairi, C.B. Behravesh, P. Bilivogui, S.A. Bukachi, N. Casas, N. Cediel Becerra, et al. 2022e. One Health: A new definition for a sustainable and healthy future. *PLoS Pathogens* 18: e1010537. 10.1371/journal.ppat.1010537. PMC9223325 35737670

[CR19] One Health Commission. *What is One Health?* Retrieved 26.06.2026 from https://www.onehealthcommission.org/en/why_one_health/what_is_one_health/, last accessed on 26.06.2026].

[CR20] Risely, A., N. Muller-Klein, D.W. Schmid, K. Wilhelm, T.H. Clutton-Brock, M.B. Manser, and S. Sommer. 2023. Climate change drives loss of bacterial gut mutualists at the expense of host survival in wild meerkats. *Global Change Biology* 29: 5816–5828. 10.1111/gcb.16877. 37485753

[CR21] Roslund, M.I., R. Puhakka, M. Grönroos, N. Nurminen, S. Oikarinen, A.M. Gazali, O. Cinek, L. Kramná, et al. 2020. Biodiversity intervention enhances immune regulation and health-associated commensal microbiota among daycare children. *Science Advances* 6: eaba2578. 10.1126/sciadv.aba2578. PMC7556828 33055153

[CR22] Schloter, M., P. Nannipieri, S.J. Sørensen, and J.D. van Elsas. 2017. Microbial indicators for soil quality. *Biology and Fertility of Soils* 54: 1–10. 10.1007/s00374-017-1248-3.

[CR23] Schloter, M. and S. Schulz. 2025. Microbiomes at the heart of sustainable agri-food systems and global health. *Frontiers in Science* 3: 1706475. 10.3389/fsci.2025.1706475.

[CR24] Schröder, A., M. Schloter, E. Roccotiello, W.W. Weisser, and S. Schulz. 2024. Improving ecosystem services of urban soils—How to manage the microbiome of Technosols? *Frontiers in Environmental Science* 12: 1460099. 10.3389/fenvs.2024.1460099.

[CR25] Sessitsch, A., S. Wakelin, M. Schloter, E. Maguin, T. Cernava, M.-C. Champomier-Verges, C. Charles Trevor, D. Cotter Paul, et al. 2023. Microbiome interconnectedness throughout environments with major consequences for healthy people and a healthy planet. *Microbiology and Molecular Biology Reviews* 87: e00212-00222. 10.1128/mmbr.00212-22. PMC10521359 37367231

[CR26] Soliveres, S., F. van der Plas, P. Manning, D. Prati, M.M. Gossner, S.C. Renner, F. Alt, H. Arndt, et al. 2016. Biodiversity at multiple trophic levels is needed for ecosystem multifunctionality. *Nature* 536: 456–459. 10.1038/nature19092. 27533038

[CR27] UN-United Nations. 2015a. *Paris Agreement. FCCC/CP/2015/10/Add.1.*

[CR28] UN-United Nations. 2015b. *Transforming our world: The 2030 Agenda for Sustainable Development. A/RES/70/1*.

[CR29] Wasimuddin, S.D. Brandel, M. Tschapka, R. Page, A. Rasche, V.M. Corman, C. Drosten, and S. Sommer. 2018. Astrovirus infections induce age-dependent dysbiosis in gut microbiomes of bats. *ISME Journal* 12: 2883–2893. 10.1038/s41396-018-0239-1. PMC6246552 30061706

[CR30] Wasimuddin, V.M. Corman, J.U. Ganzhorn, J. Rakotondranary, Y.R. Ratovonamana, C. Drosten, and S. Sommer. 2019. Adenovirus infection is associated with altered gut microbial communities in a non-human primate. *Scientific Reports* 9: 13410. 10.1038/s41598-019-49829-z. PMC6746978 31527752

[CR31] WBGU-German Advisory Council on Global Change. 2023. *Healthy living on a healthy planet*.

